# Replacement of Fish Meal With Blends of Chicken By‐Product Meal or Tuna By‐Product Meal and Corn Protein Sources in Diets Supplemented With Jack Mackerel Meal: Impacts on Growth Performance, Feed Utilization, and Biochemical Composition of Olive Flounder (*Paralichthys olivaceus*)

**DOI:** 10.1155/anu/7947942

**Published:** 2026-08-11

**Authors:** Seong Woo Shin, Md. Rabiul Islam, Sung Hwoan Cho, Taeho Kim

**Affiliations:** ^1^ Ocean Science and Technology School, National Korea Maritime and Ocean University, Busan 49112, Republic of Korea, kmou.ac.kr; ^2^ Department of Aquaculture, Gazipur Agricultural University, Gazipur 1706, Bangladesh; ^3^ Division of Convergence on Marine Science, National Korea Maritime and Ocean University, Busan 49112, Republic of Korea, kmou.ac.kr; ^4^ Department of Marine Production Management, Chonnam National University, Yeosu 59626, Republic of Korea, jnu.ac.kr

**Keywords:** combined animal by-product and plant protein sources, feed stimulant, fish meal replacement, jack mackerel meal, *Paralichthys olivaceus*

## Abstract

This experiment evaluates impacts of substituting different levels of fish meal (FM) with combinations of animal by‐product and plant protein sources in diets supplemented with jack mackerel meal (JMM) on the growth, feed utilization, and biochemical composition of olive flounder (*P. olivaceus*). A three‐way ANOVA (two animal by‐product sources [chicken by‐product meal (CBM) or tuna by‐product meal (TBM)] × 2 plant protein sources [corn gluten meal (CGM) or corn protein concentrate (CPC)] × 2 levels of FM replacement [25% and 50%]) model was employed. The control (Con) diet included 60% FM. In the Con diet, 25% and 50% of FM were replaced with combinations of animal by‐product (CBM or TBM) and plant (CGM or CPC) protein sources, and then 12% JMM was included as feed enhancer at the expense of FM. These experimental diets were named as the CBCG25, CBCG50, TBCG25, TBCG50, CBCP25, CBCP50, TBCP25, and TBCP50 dies, respectively. All diets were assigned to triplicate groups of fish. Six hundred and seventy‐five juvenile (initial body weight of 4.25 g) were dispersed into 27 of 50 L tanks. Fish were hand‐fed to satiation twice daily for 8 weeks. The TBM‐containing diets exhibited significantly (*p* < 0.001, *p* < 0.006, and *p* < 0.005, respectively) higher weight gain (WG), specific growth rate (SGR), and feed consumption (FC) of fish than the CBM‐containing diets. Dietary higher (50%) FM replacement levels led to significantly (*p* < 0.001 for all) lower WG, SGR, and FC than dietary lower (25%) FM replacement levels. WG of fish fed the TBCG25 and TBCP25 diets was significantly (*p* < 0.001) higher than that of fish fed the CBCG50, TBCG50, CBCP50, and TBCP50 diets. SGR of fish fed the TBCG25 diet was significantly (*p* < 0.001) higher than that of fish fed the CBCG50, TBCG50, CBCP50, and TBCP50 diets. Fish fed the TBCG25 and TBCP25 diets led to significantly (*p* < 0.002) higher FC than those fed the CBCG50 and CBCP50 diets. Feed utilization, blood chemistry, and biochemical composition of fish, with exception of fatty acid (FA) profiles, were unaffected by dietary treatments. Conclusively, the TBCG25 and TBCP25 diets seems to be most viable option for optimizing WG, SGR, and FC.

## 1. Introduction

Global aquaculture is primarily driven by finfish production, which relies heavily on formulated feeds [1]. The global usage of formulated diets in aquaculture operations was approximately 51.2 million metric tons (MT) in 2017, and it is projected to climb to 73.2 million MT by 2026 [2]. Feed costs typically represent the most significant portion of aquaculture operating expenses, accounting for 60%–70% of total costs [3]. Protein is the most costly and vital nutrient required for the growth and physiological maintenance of aquatic animals [4]. Fish meal (FM) has commonly been the gold standard protein source due to its high protein content, balanced amino acid (AA) and fatty acid (FA) profiles, excellent palatability, and high digestibility [5]. Nevertheless, the international market price for FM has surged due to rising global demand, limited wild stocks, and the rapid expansion of the aquaculture sector [6]. Notably, over the last two decades, global FM production has declined by 26.5%, while its market price has soared approximately 3.5‐fold [7]. This upward trend is expected to persist, further inflating both feed and overall production expenditures. Consequently, identifying cost‐effective and stably available protein alternatives to FM is an urgent necessity for the sustainable development of the aquaculture industry.

Plant‐derived protein sources have been considered promising substitutes for FM due to their lower price and greater availability compared with animal‐derived protein sources; however, their extensive use is limited by imbalanced AA profiles, the presence of anti‐nutritional factors (ANFs), and higher fiber content [4]. Nevertheless, advanced processing techniques have been developed and increased the protein level in plant by‐products, while reducing ANFs and fiber content [8]. Plant protein concentrates, such as corn gluten meal (CGM) and corn protein concentrate (CPC), are such by‐products obtained from the wet milling of corn [9]. CGM and CPC have considerable potential as partial replacers for FM in aquafeeds because of their low fiber content, near‐complete removal of ANFs, and high protein content (60%–80% of dry matter) [5]. Although CGM contains relatively low levels of lysine and arginine [10], it has been reported to successfully replace a portion of FM in feeds of rose snapper (*Lutjanus guttatus*) [11] and Asian seabass (*Lates calcarifer*) [12]. In addition, Kikuchi [13] showed that up to 40% of FM could be replaced with CGM in the diet of olive flounder. It has also been reported that CPC could replace FM by up to 25.2% and 53.4% in rainbow trout (*Oncorhynchus mykiss*) [14] and Nile tilapia (*Oreochromis niloticus*) [15] diets, respectively, without impairing growth. However, higher levels of FM replacement with CGM or CPC are constrained by imbalances in essential AA (EAA) and FA (EFA) profiles.

In aquafeeds, plant‐derived protein sources are generally less effective than animal‐derived protein sources [16]. Animal by‐product meals, such as tuna by‐product meal (TBM) and chicken by‐product meal (CBM), which are produced from by‐products generated in the tuna canning and chicken processing industries, respectively, have been recognized as effective alternatives to FM [17, 18]. In previous studies, TBM has been evaluated as a novel ingredient for partially substituting FM in diets of olive flounder [17, 19, 20], rockfish (*Sebastes schlegeli*) [18, 21], rose snapper [22], and red sea bream (*Pagrus major*) [23]. Similarly, CBM has also been tested as a FM replacer in olive flounder [24] and red sea bream [25] feeds. For example, Ha et al. [26] reported that up to 50% of FM could be replaceable with CBM in diet without impairing the growth and feed utilization of olive flounder. Moreover, Kim et al. [17] proved that TBM and CBM could substitute 30% of FM without adversely influencing growth and feed utilization when 30% of FM was substituted with various animal proteins, such as TBM, CBM, meat and bone meal, meat meal, hydrolyzed chicken offal meal, and blood meal, in a 65% FM‐basal feed of olive flounder. However, imbalances in dietary EAAs, particularly methionine and lysine in CBM and TBM, limit their inclusion at high levels as deficiencies in these AAs suppress fish growth [20, 27].

Taken together, these findings indicate that both plant‐ and animal‐derived protein sources have inherent limitations compared with FM in aquafeeds. Thus, strategic combination of plant and animal protein sources may represent an effective and sustainable approach for replacing expensive FM while achieving a nutritionally balanced diet [16]. However, reduced feed consumption (FC) due to lower palatability is a common concern when FM is substituted with alternative protein sources in aquafeeds [28]. Ng et al. [29] highlighted that the inclusion of feed enhancers in practical diets could improve the FC in fish. In this regard, jack mackerel meal (JMM) has been reported to function effectively as a feed enhancer in diets of olive flounder [30], rockfish [31], and yellowtail (*Seriola quinqueradiata*) [32], thereby improving growth performance and FC. JMM has also been reported to be the most attractive for feeding by rockfish and olive flounder among 15–16 typical crude protein ingredients [33, 34]. Moreover, Ikeda et al. [35] and Takakuwa et al. [36] demonstrated that histidine, nucleotides, and inosine monophosphate (IMP) present in jack mackerel muscle extracts exerted strong stimulatory impacts on feeding behavior in olive flounder and greater amberjack (*Seriola dumerili*). In addition, Jeong et al. [37] reported that the inclusion of 12% JMM in a 60% FM‐basal diet yielded the greatest weight gain (WG) and highest FC in olive flounder.

In the Republic of Korea, olive flounder was the most highly farmed marine finfish species in aquaculture in 2024, with a total production of 40,124 MT [38]. Its popularity can be attributed to its favorable taste, resistance to disease, adaptability to various environmental conditions, and high market demand [39]. As a carnivorous species, olive flounder necessitates a high‐protein diet (up to 60%), which traditionally relies on FM. However, the rising costs and supply fluctuations of FM have prompted researchers to explore sustainable protein alternatives [40]. While Choi et al. [41, 42] previously investigated the efficacy of blended animal and plant protein sources, such as poultry by‐product meal, tankage meal, and SPC, as a FM substitute for this species, there is still a significant knowledge gap. Specifically, limited research has evaluated the combined effects of these alternative protein sources in diets supplemented with JMM as a palatability enhancer.

Accordingly, this experiment was executed to evaluate the impacts of replacing different levels of FM with combinations of animal by‐product (TBM and CBM) and plant (CGM and CPC) protein sources in diets supplemented with JMM as a feed enhancer on growth, feed intake, feed utilization, biochemical composition, and blood chemistry of olive flounder.

## 2. Materials and Methods

### 2.1. Experimental Diet Preparation

A three‐way ANOVA (two animal by‐product sources [CBM or TBM] × two plant protein sources [CGM or CPC] × two levels of FM replacement [25% and 50%]) model was employed to investigate the impacts of low‐FM diets on the growth and feed availability of fish. Nine experimental diets were formulated to be isoproteic (51.5%) and isolipidic (11.5%) (Table 1). The control (Con) diet included 60% FM and 15% fermented soybean meal as the primary protein sources, with wheat flour and fish oil serving as the carbohydrate and lipid sources, respectively. In the Con diet, 25% and 50% of FM were replaced with combinations of animal by‐product meal (CBM or TBM) and plant protein source (CGM or CPC) at a 1:1 ratio, and then, 12% JMM was included as a palatability enhancer at the expense of FM, based on Jeong et al. [37], in which 12% JMM in a 60% FM‐basal diet led to the greatest WG and highest FC for olive flounder. These experimental diets were named CBCG25, CBCG50, TBCG25, TBCG50, CBCP25, CBCP50, TBCP25, and TBCP50 diets, respectively. All experimental diets were assigned to triplicate groups of fish.

**Table 1:** Ingredient and chemical composition of the experimental diets (%, DM basis).

	Experimental diets								
	Con	CBCG25	CBCG50	TBCG25	TBCG50	CBCP25	CBCP50	TBCP25	TBCP50
Ingredients (%, DM)									
Fish meal (FM)^a^	60.00	33.00	18.00	33.00	18.00	33.00	18.00	33.00	18.00
Chicken by‐product meal (CBM)^b^	—	7.40	14.80	—	—	6.70	13.40	—	—
Tuna by‐product meal (TBM)^c^	—	—	—	7.50	15.00	—	—	6.90	13.80
Corn gluten meal (CGM)^d^	—	7.40	14.80	7.50	15.00	—	—	—	—
Corn protein concentrate (CPC)^e^	—	—	—	—	—	6.70	13.40	6.90	13.80
Jack mackerel meal (JMM)^f^	—	12.00	12.00	12.00	12.00	12.00	12.00	12.00	12.00
Fermented soybean meal	15.00	15.00	15.00	15.00	15.00	15.00	15.00	15.00	15.00
Wheat flower	20.50	18.90	17.90	18.40	16.90	20.50	21.10	19.80	19.80
Fish oil	2.00	3.80	5.00	4.10	5.60	3.60	4.60	3.90	5.10
Vitamin premix^g^	1.00	1.00	1.00	1.00	1.00	1.00	1.00	1.00	1.00
Mineral premix^h^	1.00	1.00	1.00	1.00	1.00	1.00	1.00	1.00	1.00
Choline	0.50	0.50	0.50	0.50	0.50	0.50	0.50	0.50	0.50
Nutrients (%, DM)									
Dry matter	97.04	96.72	96.46	96.83	96.72	96.58	96.34	96.56	96.34
Crude protein	51.72	51.78	51.75	51.80	51.64	51.64	51.76	51.69	51.70
Crude lipid	11.55	11.53	11.51	11.65	11.60	11.72	11.58	11.63	11.61
Ash	11.84	9.83	8.88	10.08	9.26	9.79	8.63	9.41	8.91

^a^Fish meal (crude protein: 70.1%, crude lipid: 8.5%, and ash: 16.6%) was imported from Peru (USD 1.81/kg FM, USD 1 = 1,218 KRW [Korean currency]).

^b^Chicken by‐product meal (CBM) (crude protein: 65.9%; crude lipid: 11.3%, and ash: 13.6%) was purchased from Chamfre Co., Ltd., Buan‐gun, Jeollabuk‐do, Republic of Korea (USD 0.94/kg CBM).

^c^Tuna by‐product meal (TBM) (crude protein: 63.1%, crude lipid: 8.5%, and ash: 20.3%) was purchased from Woojin Feed Ind. Co., Ltd. (Incheon, Republic of Korea) (USD 1.30/kg TBM).

^d^Corn gluten meal (CGM) (crude protein: 69.4%, crude lipid: 1.0%, and ash: 2.5%) was purchased from Hyunjin Farm, Busan, Republic of Korea (USD 0.60/kg CGM).

^e^Corn protein concentrate (CPC) (crude protein: 79.3%, crude lipid: 2.8%, and ash: 1.0%) was purchased from Thefeed Co., Ltd., Busan Metropolitan City, Republic of Korea (USD 1.20/kg CPC).

^f^Jack mackerel meal (crude protein: 72.20%, crude lipid: 9.88%, and ash: 14.25%) was imported from Chile (USD 2.67/kg JMM).

^g^Vitamin premix contained the following substances diluted in cellulose (g/kg mix): L, ascorbic acid; 121.2; DL‐α‐tocopheryl acetate, 18.8; thiamin hydrochloride, 2.7; riboflavin, 9.1; pyridoxine hydrochloride, 1.8; niacin, 36.4; Ca‐D, pantothenate; 12.7; myo‐inositol, 181.8; D, biotin; 0.27; folic acid, 0.68; p‐aminobenzoic acid, 18.2; menadione, 1.8; retinyl acetate, 0.73; cholecalciferol, 0.003; and cyanocobalamin, 0.003.

^h^Mineral premix contained the following ingredients (g/kg mix): MgSO_4_ · 7H_2_O, 80.0; NaH_2_PO_4_ · 2H_2_O, 370.0; KCl, 130.0; ferricitrate, 40.0; ZnSO_4_ · 7H_2_O, 20.0; Ca‐lactate, 356.5; CuCl, 0.2; AlCl_3_·6H_2_O, 0.15; KI, 0.15; Na_2_Se_2_O_3_, 0.01; MnSO_4_·H_2_O, 2.0; and CoCl_2_ · 6H_2_O, 1.0.

All ingredients of each diet were thoroughly mixed, after which water was added at a 3:1 ratio to form a dough using a vertical mixer (B20GA; Eben Commerce Korea, Ansan‐si, Gyeonggi‐do, Korea). The dough was then pelletized (4–6 mm in diameter) using a pellet extruder (SMC‐32; SL Company, Incheon City, Korea). The pellets were then dried in a forced air oven (JW‐1350ED; Jinwoo Electronics Co., Ltd., Hwaseong‐si, Gyeonggi‐do, Korea) at 30°C for 48 h and kept at −20°C until use. All experimental diets were hand‐fed to apparent satiation twice (08:00 and 17:00) a day for 8 weeks.

### 2.2. Experimental Conditions

Healthy juveniles of similar size were acquired from a commercial hatchery and transferred to the laboratory. Fish were acclimated to the experimental conditions for 2 weeks by providing commercial pellets (52% crude protein and 12% crude lipid) two times a day at 2%–3% biomass. Six hundred and seventy‐five juveniles (initial body weight of 4.25 ± 0.03 g; mean ± SE) were randomly distributed into 27 of 50 L flow‐through tanks (25 fish/tank). The sand‐filtered seawater was supplied into each tank at a ratio of 4.2 L/min, and proper aeration was supplied constantly. The daily monitoring of water quality was conducted using a multipurpose water quality meter. The temperature, dissolved oxygen, salinity, and pH were in the ranges of 12.5–20.0°C (16.3 ± 2.38°C; mean ± SD), 7.0–8.8 mg/L (8.0 ± 0.52 mg/L), 30.3–33.6 g/L (32.2 ± 0.68 g/L), and 6.1–7.6 (6.9 ± 0.41), respectively.

Each of the nine experimental diets was assigned to triplicate groups of fish. Fish were cautiously hand‐fed to apparent satiation twice daily (08:00 and 17:00) throughout the 56‐day feeding study. The bottoms of the tanks were siphon‐cleaned daily. Uneaten feed was not collected.

### 2.3. Measurement of Growth, Feed Utilization, and Biological Indices

After the completion of the 56‐day feeding experiment, all live fish were unfed for 24 h and then anesthetized with tricaine methanesulfonate (MS‐222) at a concentration of 100 mg/L. Live fish from each tank were counted to calculate the survival, and their collective weight was measured to determine the WG. Ten anesthetized fish that were randomly selected from each tank were individually weighed, measured for their total length, and then dissected to collect the viscera and liver in order to calculate their viscerosomatic index (VSI) and hepatosomatic index (HSI). The growth performance, feed utilization, and biological indices of fish were calculated the same as those of Jeong et al. [37]’s study.

### 2.4. Analysis of the Biochemical Composition of the Samples

Fifteen fish at the beginning of the experiment and all fish after the measurements of biological indices of fish from each tank were homogenized and used for the chemical biochemical composition analysis. The formulated diets and experimental fish were subjected to chemical analysis following the AOAC standard procedure [43]. To analyze AAs, apart from methionine and cysteine, formulated diets and the whole‐body fish were hydrolyzed with 6 N HCl for 24 h at 110°C, followed by ion exchange chromatography with an AA analyzer (L‐8800 Auto‐analyzer: Hitachi, Tokyo, Japan). Lipids for FA analyses in diets and fish were extracted using a mixture of chloroform and methanol (2:1 v/v), in line with the method of Folch et al. [44]. FA methyl esters were prepared by transesterification with 14% BF_3_‐MeOH and analyzed by gas chromatography (Trace GC; Thermo, Waltham, MA, USA). For the chemical composition, AA, and FA analyses, the methods and procedures employed in the study by Jeong et al. [37] were used.

### 2.5. Analysis of Blood Chemistry

Blood was drawn by using heparinized syringes from the caudal veins of five anesthetized fish from each tank. The plasma was then extracted and stored in separate aliquots in a freezer at −70°C after centrifugation (2716 × *g* at 4°C) for 10 min. An automated chemistry system (Fuji Dri‐Chem NX500i; Fujifilm, Tokyo, Japan) was used to analyze alanine transaminase (ALT), aspartate transaminase (AST), alkaline phosphatase (ALP), total bilirubin (T‐BIL), total cholesterol (T‐CHO), triglyceride (TG), total protein (TP), and albumin (ALB).

Blood was also drawn by using syringes from five anesthetized fish from each tank. The serum was extracted and stored in separate aliquots in a freezer at −70°C after centrifugation (2716 × *g* at 4°C) for 10 min. Serum lysozyme activity was measured using the turbidimetric assay in accordance with the procedure reported by Lange et al. [45], and superoxide dismutase (SOD) was measured using a commercial SOD Assay kit (Sigma MBS705758; Sigma, St. Louis, MO, USA), in line with the manufacturer’s protocols.

### 2.6. Statistical Analysis

The significance of between‐group differences in means was examined using three‐way ANOVA and Tukey’s post hoc test after performing tests on the normality (Shapiro–Wilk’s test) and homogeneity (Levene’s test) of the data using SPSS version 24.0 (SPSS Inc., Chicago, IL, USA). Before statistical analysis, the arcsine transformation was applied to the percentage data. Statistical significance was set at *p*  < 0.05.

## 3. Results

### 3.1. AA and FA Profiles in the Formulated Feeds

All EAAs and non‐EAAs (NEAAs) exhibited relatively high levels in JMM compared to FM (Table 2). Animal by‐product sources were rich in arginine, lysine, threonine, and tryptophan among the EAAs, while plant protein sources were abundant in leucine, methionine, and phenylalanine. Increased substitution levels of FM by combination of animal by‐product and plant protein sources in diets resulted in increases in leucine and phenylalanine but decreases in arginine, lysine, methionine, threonine, tryptophan, and valine among EAAs. Increased substitution levels of FM by combination of animal by‐product and plant protein sources in diets led to increases in cysteine, glutamic acid, and proline among EAAs. Glutamic acid was the most prevalent among the NEAAs in all experimental feeds.

**Table 2:** Amino acid profile (% of diet) of the feed ingredients and experimental diets.

	Ingredients							Experimental diets								
	FM	JMM	TBM	CBM	CGM	CPC	Requirements	Con	CBCG25	CBCG50	TBCG25	TBCG50	CBCP25	CBCP50	TBCP25	TBCP50
Essential amino acids (EAAs, %)																
Arginine	4.02	4.19	3.53	4.14	2.15	1.99	2.04–2.10^a^	3.00	2.99	2.60	2.78	2.50	2.89	2.55	2.71	2.41
Histidine	1.61	3.03	1.88	1.29	1.32	1.35	—	1.35	1.29	1.24	1.42	1.27	1.30	1.25	1.43	1.28
Isoleucine	2.48	2.90	2.41	1.95	2.44	2.07	—	2.16	2.17	2.00	2.25	2.21	2.12	1.97	2.19	2.10
Leucine	5.23	5.29	4.35	4.08	10.95	12.7	—	3.80	4.17	4.50	4.25	4.53	4.45	4.68	4.47	4.79
Lysine	5.53	5.96	4.54	3.92	1.22	0.93	1.55–2.16^b^	3.78	3.15	2.81	3.31	2.90	3.13	2.79	3.18	2.81
Methionine	2.01	2.10	1.39	1.27	1.75	1.80	1.44–1.49^c^	1.32	1.23	1.18	1.26	1.22	1.25	1.19	1.27	1.23
Phenylalanine	2.77	2.86	2.38	2.22	4.02	4.48	—	2.17	2.18	2.23	2.19	2.28	2.21	2.33	2.22	2.35
Threonine	3.14	3.28	2.57	2.43	2.28	2.42	1.03^d^	1.91	1.70	1.57	1.85	1.62	1.87	1.63	1.90	1.67
Tryptophan	0.45	1.22	0.46	0.47	0.28	0.29	—	0.41	0.53	0.49	0.50	0.45	0.53	0.48	0.52	0.48
Valine	3.04	3.54	2.99	2.58	2.92	2.57	—	2.87	2.94	2.84	3.01	2.97	2.86	2.71	2.90	2.81
∑EAAs^e^	30.28	34.37	26.50	24.35	29.33	30.60	—	22.77	22.35	21.46	22.82	21.95	22.61	21.58	22.79	21.93
Non‐essential amino acids (NEAAs, %)																
Alanine	4.53	4.57	3.97	4.31	4.67	5.67	—	3.04	3.03	3.00	3.00	2.97	3.17	3.24	3.11	3.18
Aspartic acid	6.40	6.69	5.21	4.87	3.22	3.74	—	4.80	4.46	4.30	4.50	4.35	4.53	4.38	4.57	4.42
Cysteine	0.86	0.90	0.65	0.75	1.30	1.70	—	0.72	0.77	0.80	0.75	0.80	0.80	0.90	0.79	0.85
Glutamic acid	8.95	9.28	7.30	8.22	11.36	13.8	—	7.48	7.60	7.77	7.62	7.67	8.00	8.43	7.74	8.20
Glycine	3.83	4.39	4.25	6.05	1.43	1.68	—	3.06	3.11	3.08	2.88	2.80	3.21	3.22	2.90	2.81
Proline	2.82	3.05	2.84	3.94	5.14	6.47	—	2.34	2.64	2.81	3.11	3.14	2.70	3.02	2.67	2.88
Serine	2.94	3.05	2.36	2.46	2.72	3.26	—	1.52	1.45	1.40	1.43	1.38	1.54	1.53	1.48	1.45
Tyrosine	2.06	2.04	1.49	1.44	2.31	2.84	—	0.68	0.65	0.60	0.66	0.63	0.73	0.77	0.75	0.78
∑NEAAs^f^	32.39	33.97	28.07	32.04	32.15	39.16	—	23.64	23.71	23.76	23.95	23.74	24.68	25.49	24.01	24.57

^a^Arginine requirement was obtained from the study of Alam et al. [46].

^b^Lysine requirement was obtained from the study of Forster and Ogata [47].

^c^Methionine requirement was obtained from the study of Alam et al. [48].

^d^Threonine requirement was obtained from the study of Hasanthi et al. [49].

^e^∑EAAs: Total essential amino acids.

^f^∑NEAAs: Total non‐essential amino acids.

Total n‐3 highly unsaturated FAs (∑n‐3 HUFAs) including eicosapentaenoic acid (EPA) (C20:5n–3) and docosahexaenoic acid (DHA) (C22:6n–3) were more abundant in FM and JMM than animal‐derived protein sources (TBM and CBM) and plant‐derived protein sources (CGM and CPC) (Table 3). Total monounsaturated FAs (∑MUFAs) were predominant in animal‐derived protein sources (TBM and CBM), whereas linoleic acid (18:2n–6) was mainly detected in plant‐derived protein sources (CGM and CPC). Neither EPA nor DHA was detected in CGM and CPC. Increased replacement levels of FM with combinations of animal‐ and plant‐derived protein sources in diets resulted in an increase in ∑MUFAs but decreases in ∑SFAs and ∑n‐3 HUFAs.

**Table 3:** Fatty acid (% of total fatty acids) profile of the feed ingredients and experimental diets.

Fatty acids	Ingredients						Experimental diets								
	FM	JMM	TBM	CBM	CGM	CPC	Con	CBCG25	CBCG50	TBCG25	TBCG50	CBCP25	CBCP50	TBCP25	TBCP50
C12:0	0.16	0.11	0.14	0.14	0.01	0.01	0.05	0.04	0.04	0.04	0.04	0.03	0.04	0.04	0.04
C14:0	6.41	5.01	4.22	0.97	0.07	0.07	4.04	3.42	3.01	3.65	3.21	3.45	3.08	3.61	3.17
C16:0	23.34	21.42	27.36	28.12	12.83	13.43	17.56	16.37	15.97	16.21	15.82	16.4	16.00	16.35	15.96
C18:0	5.01	7.44	8.06	8.71	2.12	1.86	2.85	3.47	3.51	3.40	3.43	3.45	3.46	3.37	3.36
C20:0	0.32	0.32	0.69	0.17	0.49	0.43	0.17	0.16	0.18	0.23	0.25	0.15	0.14	0.20	0.24
C24:0	2.56	3.31	1.26	0.04	0.00	0.00	0.37	0.27	0.19	0.33	0.23	0.28	0.20	0.33	0.25
∑SFA^a^	37.80	37.61	41.73	38.15	15.52	15.80	25.04	23.73	22.90	23.86	22.98	23.76	22.92	23.90	23.02
C14:1n‐5	0.13	0.14	0.05	0.24	0.00	0.00	0.09	0.09	0.10	0.07	0.06	0.08	0.10	0.07	0.06
C16:1n‐7	6.80	6.81	5.78	6.05	0.25	0.24	5.58	5.02	4.37	4.87	4.32	5.03	4.41	4.84	4.29
C18:1n‐9	16.04	21.22	24.22	48.21	25.93	24.57	23.03	27.35	30.34	26.67	28.22	27.10	29.60	26.11	27.37
C20:1n‐9	4.17	1.80	2.30	1.05	0.01	0.01	3.97	3.07	2.00	3.15	2.45	3.05	1.97	3.14	2.43
C22:1n‐9	0.45	0.83	2.16	0.06	0.00	0.00	3.27	3.14	3.10	3.43	3.60	3.12	3.07	3.43	3.57
∑MUFA^b^	27.59	30.80	34.51	55.61	26.19	24.82	35.94	38.67	39.91	38.19	38.65	38.38	39.15	37.59	37.72
C18:2n‐6	4.17	1.73	2.37	4.86	53.66	54.03	21.34	23.67	26.67	23.17	25.58	24.15	28.24	23.34	26.00
C18:3n‐3	0.57	0.76	0.80	0.06	2.51	2.54	2.84	3.01	3.05	3.25	3.40	3.05	2.99	3.21	3.43
C18:3n‐6	0.23	0.10	0.32	0.02	0.01	0.01	0.09	0.06	0.05	0.07	0.06	0.06	0.06	0.08	0.08
C20:2n‐6	0.08	0.17	0.29	0.08	0.05	0.05	0.14	0.14	0.13	0.17	0.21	0.13	0.13	0.17	0.20
C20:3n‐3	0.08	0.00	0.00	0.00	0.03	0.03	0.06	0.05	0.03	0.05	0.03	0.05	0.04	0.05	0.03
C20:3n‐6	0.10	0.00	0.00	0.00	0.00	0.01	0.06	0.06	0.05	0.06	0.05	0.05	0.05	0.05	0.04
C20:4n‐6	1.14	0.69	1.31	0.36	0.00	0.00	0.26	0.20	0.18	0.23	0.26	0.20	0.17	0.22	0.26
C20:5n‐3	10.54	10.73	4.58	0.13	0.00	0.00	5.59	3.87	2.79	4.36	3.49	3.63	2.69	4.33	3.35
C22:6n‐3	13.30	15.40	10.48	0.37	0.00	0.00	6.34	5.01	3.84	5.93	4.89	4.88	3.38	5.46	4.47
∑n‐3 HUFA^c^	23.92	26.13	15.06	0.50	0.03	0.03	11.99	8.93	6.66	10.34	8.41	8.56	6.11	9.84	7.85
Unknown	4.40	2.01	3.61	0.36	2.03	2.72	2.30	1.53	0.40	0.66	0.40	1.66	0.18	1.60	1.40

^a^∑SFA: Total saturated fatty acids.

^b^∑MUFA: Total monounsaturated fatty acids.

^c^∑n‐3 HUFA: Total n‐3 highly unsaturated fatty acids.

### 3.2. Growth Performance, Feed Utilization, and Biological Indices of Fish

Fish survival exceeded 96%, but neither dietary animal by‐product sources, plant protein sources, nor FM replacement levels significantly (*p* > 0.05) affected survival (Table 4). The TBM‐containing diets exhibited significantly higher WG and specific growth rate (SGR) than the CBM‐containing diets (*p* < 0.001 and *p* < 0.006, respectively). Dietary higher (50%) FM replacement levels led to significantly (*p* < 0.001 for all) lower WG and SGR than dietary lower (25%) FM replacement levels. Olive flounder fed the TBCG25 and TBCP25 diets exhibited statistically (*p* < 0.001) higher WG than those fed the CBCG50, TBCG50, CBCP50, and TBCP50 diets, but not statistically (*p* > 0.05) different from those fed the Con, CBCG25, and CBCP25 diets. SGR of olive flounder fed the TBCG25 diet was significantly (*p* < 0.001) higher than that of fish fed the CBCG50, TBCG50, CBCP50, and TBCP50 diets, but not significantly (*p* > 0.05) different from that of fish fed the Con, CBCG25, CBCP25, and TBCP25 diets. The greatest WG and SGR were achieved in fish fed the TBCG25 diet.

**Table 4:** Survival (%), weight gain (WG, g/fish), and specific growth rate (SGR, %/day) of olive flounder (*Paralichthys olivaceus*) fed the experimental diets for 56 days.

Experimental diets	Initial weight (g/fish)	Final weight (g/fish)	Survival (%)	WG (g/fish)	SGR^1^ (%/day)
Con	4.17 ± 0.04	19.66 ± 0.21^ab^	98.67 ± 1.33	15.49 ± 0.19^ab^	2.77 ± 0.01^ab^
CBCG25	4.23 ± 0.05	19.71 ± 0.21^ab^	98.67 ± 1.33	15.48 ± 0.17^ab^	2.75 ± 0.00^abc^
CBCG50	4.12 ± 0.03	18.35 ± 0.21^c^	96.00 ± 2.30	14.23 ± 0.18^c^	2.67 ± 0.01^cd^
TBCG25	4.23 ± 0.03	20.31 ± 0.07^a^	97.33 ± 1.33	16.07 ± 0.08^a^	2.80 ± 0.02^a^
TBCG50	4.28 ± 0.02	19.27 ± 0.05^bc^	98.67 ± 1.33	15.00 ± 0.06^bc^	2.70 ± 0.01^bcd^
CBCP25	4.26 ± 0.05	19.81 ± 0.08^ab^	97.33 ± 1.33	15.53 ± 0.11^ab^	2.74 ± 0.02^abcd^
CBCP50	4.25 ± 0.03	18.72 ± 0.09^c^	96.00 ± 2.30	14.45 ± 0.12^c^	2.64 ± 0.02^d^
TBCP25	4.24 ± 0.03	20.26 ± 0.35^a^	98.67 ± 1.33	16.01 ± 0.37^a^	2.79 ± 0.03^ab^
TBCP50	4.24 ± 0.04	19.13 ± 0.14^bc^	98.67 ± 1.33	14.90 ± 0.12^bc^	2.69 ± 0.01^bcd^
*p*‐Value	0.1	0.001	0.8	0.001	0.001
Main effect: AP					
CBM	—	19.15^B^	97.00	14.92^B^	2.70^B^
TBM	—	19.74^A^	98.33	15.49^A^	2.74^A^
Main effect: PP					
CGM	—	19.40	97.67	15.19	2.73
CPC	—	19.48	97.67	15.22	2.71
Main effect: RL					
25%	—	20.02^A^	98.00	15.77^A^	2.77^A^
50%	—	18.87^B^	97.33	14.64^B^	2.67^B^
Three‐way ANOVA					
AP	—	0.001	0.265	0.001	0.006
PP	—	0.594	0.999	0.832	0.440
RL	—	0.001	0.572	0.001	0.001
AP × PP	—	0.232	0.572	0.404	0.661
AP × RL	—	0.584	0.265	0.776	0.654
PP × RL	—	0.372	0.999	0.788	0.987
AP × PP × RL	—	0.489	0.572	0.662	0.681

*Note:* Values (means of triplicate ± SE) in the same column sharing the same superscript letter are not significantly different (*p* > 0.05).

Abbreviations: AP, animal by‐product protein; PP, plant protein; RL, replacement level.

^1^SGR (%/day) = (Ln final weight of fish – Ln initial weight of fish) × 100/days of feeding trial (56 days).

The TBM‐containing feeds led to significantly (*p* < 0.005) higher FC than the CBM‐containing feeds (Table 5). Dietary higher FM replacement levels exhibited significantly (*p* < 0.001) lower FC than dietary lower FM replacement levels. FC of fish fed the TBCG25 and TBCP25 diets was statistically (*p* < 0.002) higher than that of fish fed the CBCG50 and CBCP50 diets, but not statistically (*p* > 0.05) different from that of fish fed the Con, CBCG25, TBCG50, CBCP25, and TBCP50 diets.

**Table 5:** Feed consumption (g/fish), feed efficiency (FE), protein efficiency ratio (PER), protein retention (PR, %), condition factor (CF, g/cm^3^), viscerosomatic index (VSI, %), and hepatosomatic index (HSI, %) of olive flounder fed the experimental diets for 8 weeks.

Experimental diets	Feed consumption (g/fish)	FE^1^	PER^2^	PR^3^ (%)	CF^4^ (g/cm^3^)	VSI^5^ (%)	HSI^6^ (%)
Con	16.37 ± 0.18^ab^	0.95 ± 0.01	1.83 ± 0.01	30.35 ± 0.54	0.98 ± 0.01	4.32 ± 0.09	1.62 ± 0.11
CBCG25	16.00 ± 0.64^ab^	0.96 ± 0.04	1.87 ± 0.08	31.38 ± 1.43	0.98 ± 0.01	4.58 ± 0.10	1.76 ± 0.03
CBCG50	14.85 ± 0.73^b^	0.95 ± 0.06	1.85 ± 0.06	31.62 ± 0.95	0.97 ± 0.00	4.77 ± 0.23	1.73 ± 0.15
TBCG25	16.93 ± 0.33^a^	0.95 ± 0.01	1.83 ± 0.01	31.43 ± 2.33	0.98 ± 0.00	4.44 ± 0.04	1.75 ± 0.10
TBCG50	15.87 ± 0.12^ab^	0.95 ± 0.04	1.83 ± 0.02	30.48 ± 0.43	0.98 ± 0.00	4.41 ± 0.18	1.74 ± 0.10
CBCP25	16.27 ± 0.74^ab^	0.96 ± 0.01	1.85 ± 0.07	31.47 ± 1.60	0.98 ± 0.00	4.52 ± 0.06	1.65 ± 0.09
CBCP50	14.95 ± 0.25^b^	0.96 ± 0.01	1.86 ± 0.04	31.46 ± 0.89	0.97 ± 0.00	4.87 ± 0.09	1.75 ± 0.10
TBCP25	16.88 ± 0.51^a^	0.95 ± 0.01	1.83 ± 0.02	31.16 ± 0.68	0.99 ± 0.00	4.26 ± 0.11	1.72 ± 0.08
TBCP50	15.72 ± 0.06^ab^	0.95 ± 0.01	1.83 ± 0.02	30.97 ± 0.76	0.97 ± 0.00	4.53 ± 0.24	1.65 ± 0.18
*p*‐Value	0.002	0.9	0.9	0.8	0.07	0.1	0.9
Main effect: AP							
CBM	15.52^B^	0.96	1.86	31.49	0.97	4.69^A^	1.72
TBM	16.35^A^	0.95	1.83	32.01	0.98	4.41^B^	1.71
Main effect: PP							
CGM	15.91	0.96	1.85	31.23	0.98	4.55	1.75
CPC	15.96	0.96	1.85	31.27	0.98	4.55	1.69
Main effect: RL							
25%	16.52^A^	0.96	1.85	31.36	0.98^A^	4.45	1.72
50%	15.34^B^	0.95	1.85	31.13	0.97^B^	4.65	1.72
Three‐way ANOVA							
AP	0.005	0.291	0.308	0.326	0.057	0.021	0.891
PP	0.873	0.977	0.968	0.936	0.624	0.977	0.514
RL	0.001	0.913	0.936	0.640	0.029	0.091	0.984
AP × PP	0.596	0.968	0.987	0.873	0.629	0.837	0.931
AP × RL	0.805	0.950	0.986	0.478	0.306	0.505	0.664
PP × RL	0.798	0.684	0.764	0.791	0.424	0.303	0.853
AP × PP × RL	0.942	0.778	0.761	0.594	0.243	0.756	0.567

*Note:* Values (means of triplicate ± SE) in the same column sharing the same superscript letter are not significantly different (*p* > 0.05).

Abbreviations: AP, animal by‐product protein; PP, plant protein; RL, replacement level.

^1^Feed efficiency (FE) = weight gain of fish/feed supplied.

^2^Protein efficiency ratio (PER) = weight gain of fish/protein consumption.

^3^Protein retention (PR, %) = protein gain of fish × 100/protein consumption.

^4^Condition factor (CF, g/cm^3^) = body weight of fish × 100/total length of fish (cm)^3^.

^5^Viscerosomatic index (VSI, %) = viscera weight of fish × 100/body weight of fish.

^6^Hepatosomatic index (HSI, %) = liver weight of fish × 100/total body weight of fish.

FE, PER, and PR of olive flounders were in the ranges of 0.95%–0.96%, 1.83%–1.87%, and 30.35%–31.62%, respectively. Neither dietary animal by‐product sources, plant protein sources, nor FM replacement levels significantly (*p* > 0.05) affected FE, PER, and PR.

CF, VSI, and HSI of the olive flounders were in the ranges of 0.97–0.99 g/cm^3^, 4.26%–4.87%, and 1.62%–1.76%, respectively. Dietary higher FM replacement levels resulted in significantly (*p* < 0.029) lower CF than dietary lower FM replacement levels. The CBM‐containing diets led to statistically (*p* < 0.021) higher VSI than the TBM‐containing diets. However, none of these parameters were statistically (*p* > 0.05) different among dietary treatments.

### 3.3. Chemical Composition of the Whole‐Body Fish

The whole‐body moisture, crude protein, crude lipid, and ash content of fish were in the ranges of 75.34%–76.45%, 16.19%–16.64%, 3.13%–3.31%, and 3.84%–4.22%, respectively (Table 6). Neither dietary animal by‐product sources, plant protein sources, nor FM replacement levels significantly (*p* > 0.05) affected them.

**Table 6:** Chemical composition (%, wet weight) of the whole‐body olive flounder at the end of 8‐week feeding trial.

Experimental diets	Moisture	Crude protein	Crude lipid	Ash
Con	75.61 ± 0.38	16.19 ± 0.10	3.13 ± 0.07	4.03 ± 0.08
CBCG25	76.45 ± 0.04	16.33 ± 0.11	3.14 ± 0.12	4.04 ± 0.03
CBCG50	75.86 ± 0.48	16.55 ± 0.13	3.13 ± 0.04	4.05 ± 0.07
TBCG25	76.03 ± 0.29	16.64 ± 0.09	3.31 ± 0.05	4.05 ± 0.19
TBCG50	75.90 ± 0.64	16.22 ± 0.22	3.26 ± 0.09	4.22 ± 0.06
CBCP25	75.44 ± 0.29	16.48 ± 0.18	3.14 ± 0.13	4.13 ± 0.06
CBCP50	75.56 ± 0.41	16.35 ± 0.18	3.16 ± 0.13	3.91 ± 0.11
TBCP25	75.34 ± 0.20	16.51 ± 0.19	3.15 ± 0.11	4.09 ± 0.16
TBCP50	75.53 ± 0.28	16.42 ± 0.12	3.22 ± 0.11	3.84 ± 0.09
*p*‐Value	0.5	0.5	0.9	0.5
Main effect: AP				
CBM	75.84	16.43	3.14	4.03
TBM	75.71	16.45	3.24	4.05
Main effect: PP				
CGM	76.08	16.44	3.21	4.09
CPC	75.87	16.44	3.17	4.00
Main effect: RL				
25%	75.84	16.49	3.19	4.07
50%	75.71	16.39	3.19	4.00
Three‐way ANOVA				
AP	0.642	0.871	0.240	0.830
PP	0.034	0.960	0.593	0.242
RL	0.646	0.374	0.966	0.402
AP × PP	0.818	0.793	0.470	0.402
AP × RL	0.625	0.211	0.949	0.691
PP × RL	0.316	0.994	0.608	0.060
AP × PP × RL	0.723	0.159	0.748	0.590

*Note:* Means of triplicate ± SE.

Abbreviations: AP, animal by‐product protein; PP, plant protein; RL, replacement level.

### 3.4. Plasma and Serum Parameters of Fish

Plasma AST, ALT, ALP, T‐BIL, T‐CHO, TG, TP, and ALB levels of fish were in the ranges of 85.67–96.67 U/L, 8.67–11.33 U/L, 106.00–113.66 U/L, 0.86–1.13 mg/dL, 177.00–198.00 mg/dL, 332.00–364.33 mg/dL, 3.76–4.30 g/dL, and 0.90–1.16 g/dL, respectively (Table 7). The serum lysozyme activity and SOD of fish were in the ranges of 523.33–581.66 U/mL and 2.12–2.50 ng/L, respectively. Neither dietary animal by‐product protein sources, plant protein sources, nor FM replacement levels significantly (*p* > 0.05) affected plasma or serum parameters of fish.

**Table 7:** Plasma parameters, serum lysozyme activity, and SOD of olive flounder at the end of 8‐week feeding trial.

Experimental diets	Plasma parameters								Serum parameters	
	AST (U/L)	ALT (U/L)	ALP (U/L)	T‐BIL (mg/dL)	T‐CHO (mg/dL)	TG (mg/dL)	TP (g/dL)	ALB (g/dL)	Lysozyme activity (U/mL)	SOD (ng/mL)
Con	94.33 ± 5.84	11.33 ± 1.66	109.00 ± 2.64	0.87 ± 0.08	181.67 ± 6.38	332.00 ± 13.31	4.26 ± 0.20	1.06 ± 0.06	530.75 ± 6.38	2.26 ± 13.31
CBCG25	91.66 ± 2.84	8.67 ± 1.45	109.00 ± 14.17	0.96 ± 0.08	184.67 ± 10.03	364.33 ± 18.90	3.76 ± 0.14	1.16 ± 0.08	536.66 ± 10.03	2.42 ± 18.90
CBCG50	85.67 ± 3.52	9.00 ± 1.52	110.33 ± 5.36	1.00 ± 0.20	178.00 ± 13.11	354.33 ± 14.43	4.23 ± 0.26	1.13 ± 0.16	543.33 ± 13.11	2.39 ± 14.43
TBCG25	86.33 ± 2.84	9.33 ± 1.33	110.66 ± 28.66	1.03 ± 0.14	178.00 ± 17.05	333.33 ± 10.97	3.86 ± 0.18	0.90 ± 0.15	526.66 ± 17.05	2.38 ± 10.97
TBCG50	96.67 ± 5.81	10.00 ± 0.57	106.00 ± 9.81	1.06 ± 0.12	181.33 ± 10.72	332.00 ± 9.50	4.03 ± 0.18	1.03 ± 0.12	531.66 ± 10.72	2.14 ± 9.50
CBCP25	87.67 ± 5.04	9.33 ± 1.45	112.00 ± 5.50	1.13 ± 0.12	177.00 ± 13.05	352.00 ± 18.87	3.90 ± 0.43	1.00 ± 0.05	581.66 ± 13.05	2.50 ± 18.87
CBCP50	86.33 ± 3.84	10.33 ± 0.88	113.66 ± 4.91	0.90 ± 0.14	177.00 ± 16.04	363.33 ± 17.37	4.06 ± 0.14	1.13 ± 0.12	548.33 ± 16.04	2.47 ± 17.37
TBCP25	92.00 ± 4.50	11.33 ± 1.20	108.66 ± 14.19	1.13 ± 0.12	198.00 ± 10.21	361.66 ± 13.61	3.86 ± 0.29	1.06 ± 0.08	536.66 ± 10.21	2.26 ± 13.61
TBCP50	91.33 ± 6.90	10.00 ± 2.30	108.33 ± 7.35	0.86 ± 0.06	187.33 ± 17.03	358.33 ± 6.06	4.30 ± 0.05	1.00 ± 0.10	523.33 ± 17.03	2.12 ± 6.06
*p*‐Value	0.7	0.8	0.9	0.6	0.9	0.5	0.6	0.8	0.9	0.3
Main effect: AP										
CBM	87.83	9.33	111.25	1.00	179.17	358.50	3.99	1.11	552.50	2.45
TBM	91.58	10.17	108.42	1.03	186.17	346.33	4.02	1.00	529.58	2.22
Main effect: PP										
CGM	90.08	9.25	109.00	1.02	180.50	346.00	3.96	1.06	534.58	2.33
CPC	89.33	10.25	110.67	1.00	184.83	358.83	4.03	1.05	547.50	2.30
Main effect: RL										
25%	89.42	9.67	110.08	1.07	184.41	352.83	3.85	1.03	545.42	2.39
50%	90.00	9.83	109.58	0.96	180.92	352.00	4.16	1.08	536.67	2.28
Three‐way ANOVA										
AP	0.268	0.420	0.770	0.785	0.480	0.249	0.885	0.208	0.525	0.060
PP	0.822	0.335	0.864	0.927	0.661	0.225	0.735	0.921	0.719	0.957
RL	0.861	0.871	0.959	0.247	0.723	0.936	0.088	0.620	0.807	0.167
AP × PP	0.783	0.999	0.877	0.650	0.384	0.173	0.664	0.377	0.736	0.358
AP × RL	0.212	0.626	0.837	0.927	0.986	0.885	0.961	0.921	0.898	0.327
PP × RL	0.635	0.745	0.904	0.136	0.852	0.641	0.961	0.921	0.684	0.740
AP × PP × RL	0.248	0.517	0.918	0.927	0.601	0.574	0.416	0.283	0.880	0.709

*Note:* Means of triplicate ± SE.

Abbreviations: ALB, albumin; ALP, alkaline phosphatase; ALT, alanine transaminase; AP, animal by‐product protein; AST, aspartate transaminase; PP, plant protein; RL, replacement level; SOD, superoxide dismutase; T‐BIL, total bilirubin; T‐CHO, total cholesterol; TG, triglyceride; TP, total protein.

### 3.5. AA and FA Profiles of Fish

The whole‐body AA profiles in olive flounder were not statistically (*p* > 0.05) affected by either dietary animal by‐product sources, plant protein sources, or FM replacement levels (Table 8).

**Table 8:** The whole‐body amino acid profiles (% of wet weight) of olive flounder fed the experimental diets for 8 weeks.

	Experimental diets										Main effect: AP		Main effect: PP		Main effect: RL		Three‐way ANOVA						
	Con	CBCG 25	CBCG 50	TBCG 25	TBCG 50	CBCP 25	CBCP 50	TBCP 25	TBCP 50	*p*‐Value	CBM	TBM	CGM	CPC	25%	50%	AP	PP	RL	AP × PP	AP × RL	PP × RL	AP × PP × RL
Essential amino acids (%)																							
Arginine	0.89 ± 0.03	0.88 ± 0.02	0.81 ± 0.02	0.84 ± 0.02	0.80 ± 0.02	0.87 ± 0.03	0.86 ± 0.02	0.85 ± 0.14	0.82 ± 0.10	0.9	0.85	0.83	0.83	0.85	0.86	0.82	0.607	0.706	0.433	0.999	0.945	0.731	0.810
Histidine	0.34 ± 0.02	0.35 ± 0.00	0.35 ± 0.00	0.36 ± 0.00	0.36 ± 0.00	0.35 ± 0.01	0.35 ± 0.01	0.37 ± 0.00	0.37 ± 0.01	0.7	0.35	0.36	0.35	0.36	0.36	0.36	0.051	0.398	0.715	0.544	0.903	0.715	0.715
Isoleucine	0.68 ± 0.03	0.64 ± 0.02	0.63 ± 0.01	0.72 ± 0.03	0.71 ± 0.05	0.65 ± 0.01	0.63 ± 0.01	0.69 ± 0.00	0.66 ± 0.02	0.2	0.64	0.70	0.68	0.66	0.68	0.66	0.077	0.391	0.348	0.348	0.967	0.773	0.836
Leucine	1.18 ± 0.05	1.21 ± 0.02	1.23 ± 0.01	1.24 ± 0.01	1.23 ± 0.01	1.20 ± 0.05	1.24 ± 0.08	1.21 ± 0.01	1.29 ± 0.03	0.8	1.22	1.24	1.23	1.23	1.22	1.26	0.416	0.799	0.302	0.713	0.887	0.356	0.631
Lysine	1.32 ± 0.03	1.29 ± 0.05	1.22 ± 0.18	1.31 ± 0.05	1.25 ± 0.10	1.29 ± 0.03	1.23 ± 0.06	1.29 ± 0.13	1.26 ± 0.07	0.9	1.26	1.28	1.27	1.27	1.30	1.24	0.766	0.999	0.453	0.963	0.909	0.891	0.927
Methionine	0.53 ± 0.02	0.51 ± 0.01	0.48 ± 0.06	0.52 ± 0.01	0.51 ± 0.00	0.52 ± 0.01	0.49 ± 0.01	0.52 ± 0.01	0.50 ± 0.02	0.9	0.50	0.51	0.50	0.50	0.52	0.49	0.515	0.999	0.295	0.683	0.624	0.870	0.806
Phenylalanine	0.66 ± 0.02	0.65 ± 0.05	0.65 ± 0.03	0.66 ± 0.03	0.67 ± 0.02	0.65 ± 0.03	0.67 ± 0.03	0.65 ± 0.02	0.67 ± 0.00	0.9	0.66	0.66	0.66	0.66	0.65	0.66	0.767	0.974	0.669	0.717	0.869	0.767	0.818
Threonine	0.65 ± 0.03	0.61 ± 0.02	0.58 ± 0.05	0.64 ± 0.08	0.59 ± 0.07	0.64 ± 0.04	0.59 ± 0.04	0.65 ± 0.02	0.60 ± 0.03	0.9	0.60	0.62	0.60	0.62	0.63	0.59	0.679	0.679	0.257	0.879	0.948	0.810	0.879
Tryptophan	0.07 ± 0.00	0.09 ± 0.00	0.09 ± 0.00	0.09 ± 0.00	0.08 ± 0.00	0.09 ± 0.00	0.09 ± 0.00	0.10 ± 0.00	0.09 ± 0.00	0.2	0.09	0.09	0.09	0.09	0.09	0.09	0.999	0.750	0.750	0.345	0.345	0.526	0.999
Valine	0.83 ± 0.01	0.84 ± 0.02	0.82 ± 0.02	0.86 ± 0.02	0.85 ± 0.04	0.83 ± 0.02	0.81 ± 0.05	0.85 ± 0.02	0.83 ± 0.02	0.9	0.83	0.85	0.84	0.83	0.85	0.83	0.369	0.452	0.369	0.940	0.940	0.940	0.999
Non‐essential amino acids (%)																							
Alanine	1.29 ± 0.08	1.28 ± 0.09	1.31 ± 0.06	1.28 ± 0.04	1.19 ± 0.06	1.30 ± 0.06	1.32 ± 0.10	1.25 ± 0.03	1.31 ± 0.09	0.9	1.30	1.26	1.27	1.30	1.28	1.28	0.409	0.583	0.987	0.789	0.694	0.501	0.482
Aspartic acid	1.41 ± 0.09	1.34 ± 0.07	1.24 ± 0.14	1.35 ± 0.06	1.29 ± 0.09	1.36 ± 0.04	1.31 ± 0.9	1.38 ± 0.14	1.33 ± 0.07	0.9	1.31	1.34	1.30	1.34	1.36	1.29	0.728	0.579	0.348	0.926	0.871	0.817	0.926
Cysteine	0.19 ± 0.01	0.20 ± 0.00	0.20 ± 0.02	0.19 ± 0.00	0.20 ± 0.00	0.20 ± 0.00	0.21 ± 0.00	0.20 ± 0.00	0.21 ± 0.01	0.8	0.20	0.20	0.19	0.21	0.20	0.21	0.836	0.412	0.412	0.836	0.536	0.679	0.536
Glutamic acid	2.20 ± 0.04	2.31 ± 0.12	2.29 ± 0.04	2.22 ± 0.04	2.27 ± 0.04	2.31 ± 0.09	2.42 ± 0.13	2.28 ± 0.09	2.35 ± 0.05	0.7	2.33	2.32	2.27	2.34	2.28	2.33	0.384	0.284	0.427	0.969	0.906	0.539	0.628
Glycine	1.25 ± 0.05	1.27 ± 0.02	1.25 ± 0.004	1.23 ± 0.08	1.32 ± 0.11	1.32 ± 0.02	1.32 ± 0.11	1.24 ± 0.04	1.27 ± 0.07	0.9	1.29	1.27	1.27	1.30	1.27	1.29	0.646	0.736	0.624	0.464	0.521	0.878	0.713
Proline	0.67 ± 0.03	0.71 ± 0.03	0.76 ± 0.04	0.69 ± 0.06	0.74 ± 0.03	0.75 ± 0.06	0.79 ± 0.04	0.72 ± 0.07	0.77 ± 0.07	0.7	0.75	0.75	0.75	0.76	0.72	0.77	0.591	0.382	0.230	0.846	0.948	0.948	0.948
Serine	0.62 ± 0.04	0.59 ± 0.01	0.56 ± 0.08	0.58 ± 0.09	0.57 ± 0.10	0.65 ± 0.04	0.63 ± 0.09	0.60 ± 0.04	0.59 ± 0.08	0.9	0.61	0.69	0.57	0.62	0.60	0.59	0.692	0.432	0.807	0.670	0.903	0.927	0.976
Tyrosine	0.29 ± 0.04	0.27 ± 0.04	0.25 ± 00.09	0.28 ± 0.06	0.26 ± 0.04	0.30 ± 0.05	0.32 ± 0.04	0.31 ± 0.04	0.32 ± 0.02	0.9	0.28	0.29	0.27	0.31	0.29	0.29	0.791	0.182	0.981	0.904	0.942	0.581	0.866

*Note:* Means of triplicate ± SE.

Abbreviations: AP, animal by‐product protein; PP, plant protein; RL, replacement level.

The whole‐body ∑SFAs in olive flounder was not significantly (*p* > 0.05) affected by either dietary animal by‐product sources, plant protein sources, or FM replacement levels (Table 9). However, fish fed the CBM‐containing diets led to statistically (*p* < 0.001) higher the whole‐body ∑MUFAs than fish fed the TBM‐containing diets. In addition, dietary higher FM substitution levels led to statistically (*p* < 0.001) higher the whole‐body ∑MUFAs than dietary lower FM substitution levels. Significant (*p* < 0.001 for all) interactions of AP × PP, AP × RL, PP × RL, and AP × PP × RL on the whole‐body ∑MUFAs were observed. However, the whole‐body ∑MUFAs in fish was not significantly (*p* > 0.05) affected by dietary treatments. The TBM‐ and CGM‐containing diets achieved significantly (*p* < 0.001 and *p* < 0.016) higher the whole‐body ∑n‐3 HUFA than the CBM‐ and CPC‐containing diets, respectively. In addition, dietary higher FM replacement levels led to significantly (*p* < 0.001) lower the whole‐body ∑n‐3 HUFAs than dietary lower FM replacement levels. A significant interaction (*p* < 0.011) of AP × RL on the whole‐body ∑n‐3 HUFAs was observed. The levels of EPA, DHA, and ∑n‐3 HUFAs in the whole‐body fish fed the Con diet were significantly (*p* < 0.001) higher than those of fish fed all other diets.

**Table 9:** The whole‐body fatty acid profiles (% of total fatty acids) of olive flounder fed the experimental diets for 8 weeks.

	Experimental diets										Main effect: AP		Main effect: PP		Main effect: RL		Three‐way ANOVA						
	Con	CBCG 25	CBCG 50	TBCG 25	TBCG 50	CBCP 25	CBCP 50	TBCP 25	TBCP 50	*p*‐Value	CBM	TBM	CGM	CPC	25%	50%	AP	PP	RL	AP × PP	AP × RL	PP × RL	AP × PP × RL
C12:0	0.06 ± 0.01	0.06 ± 0.00	0.05 ± 0.01	0.06 ± 0.00	0.05 ± 0.00	0.05 ± 0.01	0.05 ± 0.00	0.05 ± 0.00	0.06 ± 0.01	0.9	0.05	0.05	0.05	0.05	0.05	0.05	0.627	0.807	0.807	0.999	0.627	0.468	0.999
C14:0	4.71 ± 0.19^a^	3.01 ± 0.48^b^	2.61 ± 0.26^b^	3.66 ± 0.10^ab^	2.93 ± 0.21^b^	3.17 ± 0.45^b^	2.76 ± 0.05^b^	3.41 ± 0.49^ab^	2.82± 0.07^b^	0.005	2.97	2.87	3.05	3.04	3.31^B^	3.78^A^	0.185	0.957	0.032	0.475	0.577	0.893	0.882
C16:0	16.52 ± 0.59	16.29 ± 0.36	16.18 ± 0.48	16.23 ± 0.25	16.40 ± 0.55	16.30 ± 0.29	16.20 ± 0.49	16.27 ± 0.59	16.16 ± 0.39	0.9	16.24	16.27	16.28	16.24	16.27	16.24	0.937	0.900	0.908	0.855	0.830	0.826	0.822
C18:0	3.21 ± 0.18	3.58 ± 0.25	3.62 ± 0.15	3.49 ± 0.15	3.53 ± 0.18	3.55 ± 0.31	3.57 ± 0.18	3.46 ± 0.25	3.45 ± 0.19	0.9	3.58	3.49	3.56	3.51	3.52	3.55	0.525	0.768	0.885	0.970	0.961	0.910	0.961
C20:0	0.15 ± 0.00	0.15 ± 0.00	0.15 ± 0.00	0.16 ± 0.00	0.16 ± 0.00	0.15 ± 0.00	0.14 ± 0.00	0.16 ± 0.00	0.16 ± 0.00	0.2	0.15^B^	0.16^A^	0.16	0.15	0.16	0.16	0.017	0.549	0.841	0.323	0.549	0.323	0.841
C24:0	0.96 ± 0.08	0.94 ± 0.05	0.90 ± 0.07	0.95 ± 0.07	0.92 ± 0.04	0.94 ± 0.06	0.91 ± 0.06	0.95 ± 0.04	0.94 ± 0.05	0.9	0.92	0.94	0.93	0.93	0.94	0.92	0.674	0.848	0.518	0.909	0.909	0.909	0.969
∑SFA^1^	25.62 ± 0.58	24.03 ± 0.61	23.52 ± 0.17	24.54 ± 0.28	24.00 ± 0.39	24.16 ± 0.40	23.64 ± 0.46	24.32 ± 0.79	23.59 ± 0.58	0.1	23.84	24.11	24.02	23.92	24.26	23.68	0.453	0.794	0.122	0.542	0.873	0.888	0.902
C14:1n–5	0.15 ± 0.02	0.14 ± 0.01	0.15 ± 0.00	0.15 ± 0.00	0.15 ± 0.00	0.14 ± 0.00	0.14 ± 0.00	0.14 ± 0.00	0.15 ± 0.01	0.9	0.14	0.62	0.60	0.62	0.63	0.59	0.697	0.697	0.440	0.999	0.697	0.697	0.697
C16:1n–7	5.71 ± 0.46	5.62 ± 0.27	5.43 ± 0.23	5.61 ± 0.30	5.42 ± 0.24	5.64 ± 0.35	5.44 ± 0.19	5.60 ± 0.52	5.41 ± 0.28	0.9	5.53	5.51	5.52	5.52	5.62	5.42	0.935	0.995	0.431	0.951	0.995	0.978	0.989
C18:1n–9	26.80 ± 3.82^b^	27.10 ± 0.75^b^	29.55 ± 1.00^a^	27.02 ± 0.85^b^	27.73 ± 0.35^ab^	27.05^b^ ± 0.53^a^	28.45 ± 0.86^ab^	27.01 ± 0.50^b^	28.04 ± 0.81^ab^	0.02	28.04^A^	27.45^B^	27.84	27.64	27.45^B^	28.04^A^	0.001	0.267	0.001	0.001	0.001	0.001	0.001
C20:1n–9	4.00 ± 0.42	3.68 ± 0.26	3.23 ± 0.42	3.84 ± 0.19	3.34 ± 0.20	3.77 ± 0.22	3.18 ± 0.54	3.84 ± 0.12	3.32 ± 0.32	0.2	3.47	3.59	3.52	3.53	3.78^A^	3.27^B^	0.530	0.976	0.014	0.941	0.983	0.844	0.865
C22:1n–9	0.04 ± 0.03	0.03 ± 0.00	0.03 ± 0.00	0.05 ± 0.00	0.06 ± 0.00	0.04 ± 0.00	0.04 ± 0.00	0.05 ± 0.01	0.06 ± 0.00	0.06	0.04^B^	0.05^A^	0.04	0.05	0.05	0.05	0.001	0.212	0.855	0.585	0.212	0.855	0.855
∑MUFA^2^	36.70 ± 12.03	36.57 ± 0.83	38.39 ± 0.47	36.67 ± 0.87	36.70 ± 0.18	36.64 ± 0.17	37.25 ± 0.48	36.65 ± 0.14	36.98 ± 0.64	0.1	37.21^A^	36.75^B^	37.08	36.88	36.50^B^	37.21^A^	0.001	0.242	0.001	0.001	0.001	0.001	0.001
C18:2n–6	18.87 ± 0.33^b^	22.16 ± 1.07^a^	22.74 ± 0.67^a^	22.22 ± 0.36^a^	22.50 ± 0.51^a^	22.74 ± 0.67^a^	24.98 ± 0.18^a^	22.31 ± 0.82^a^	22.65 ± 0.57^a^	0.001	23.15	22.42	22.40	23.17	22.35	23.21	0.135	0.120	0.083	0.186	0.258	0.368	0.403
C18:3n–3	1.94 ± 0.09	2.01 ± 0.28	2.03 ± 0.32	2.08 ± 0.16	2.12 ± 0.17	2.03 ± 0.22	1.98 ± 0.26	2.06 ± 0.29	2.14 ± 0.42	0.9	2.01	2.13	2.06	2.05	2.04	2.07	0.681	0.974	0.902	0.974	0.863	0.967	0.888
C18:3n–6	0.19 ± 0.01	0.18 ± 0.01	0.18 ± 0.01	0.19 ± 0.01	0.18 ± 0.01	0.18 ± 0.01	0.18 ± 0.01	0.19 ± 0.01	0.19 ± 0.01	0.9	0.18	0.19	0.18	0.19	0.18	0.18	0.681	0.974	0.902	0.974	0.863	0.967	0.888
C20:2n–6	0.64 ± 0.04	0.64 ± 0.08	0.63 ± 0.04	0.66 ± 0.05	0.69 ± 0.03	0.63 ± 0.04	0.63 ± 0.03	0.66 ± 0.03	0.68 ± 0.04	0.9	0.63	0.67	0.66	0.65	0.65	0.66	0.323	0.820	0.785	0.927	0.682	0.999	0.891
C20:3n–3	0.24 ± 0.01	0.23 ± 0.01	0.21 ± 0.01	0.23 ± 0.01	0.21 ± 0.01	0.23 ± 0.01	0.22 ± 0.01	0.23 ± 0.01	0.21 ± 0.01	0.3	0.22	0.21	0.22	0.22	0.23^A^	0.21^B^	0.468	0.627	0.025	0.468	0.468	0.999	0.807
C20:3n–6	0.12 ± 0.01	0.12 ± 0.01	0.11 ± 0.01	0.11 ± 0.01	0.11 ± 0.01	0.11 ± 0.01	0.11 ± 0.01	0.11 ± 0.01	0.10 ± 0.01	0.9	0.11	0.11	0.11	0.11	0.11	0.11	0.613	0.613	0.450	0.800	0.613	0.613	0.450
C20:4n–6	0.65 ± 0.01	0.60 ± 0.02	0.64 ± 0.02	0.63 ± 0.08	0.66 ± 0.03	0.60 ± 0.02	0.62 ± 0.03	0.62 ± 0.05	0.65 ± 0.05	0.9	0.61	0.64	0.63	0.62	0.61	0.64	0.451	0.723	0.394	0.919	0.999	0.839	0.960
C20:5n–3	5.82 ± 0.07^a^	4.26 ± 0.04^bcd^	3.82 ± 0.08^ef^	4.64 ± 0.10^b^	4.19 ± 0.03^cde^	4.23 ± 0.09^cd^	3.75 ± 0.05^f^	4.54 ± 0.10^bc^	4.14 ± 0.06^de^	0.001	4.01^B^	4.38^A^	4.23	4.16	4.42^A^	3.98^B^	0.001	0.289	0.001	0.871	0.734	0.941	0.690
C22:6n–3	8.35 ± 0.14^a^	6.45 ± 0.11^d^	5.52 ± 0.08^e^	7.64 ± 0.05^b^	7.10 ± 0.12^c^	7.03 ± 0.06^c^	5.20 ± 0.08^e^	7.41 ± 0.06^bc^	6.25 ± 0.07^d^	0.001	6.22^B^	6.94^A^	6.68	6.48	7.30^A^	5.86^B^	0.001	0.004	0.001	0.892	0.001	0.375	0.263
∑n‐3 HUFA^3^	14.41 ± 0.19^a^	10.94 ± 0.12^de^	9.56 ± 0.13^f^	12.51 ± 0.14^b^	11.50 ± 0.16^cd^	11.50 ± 0.14^cd^	9.18 ± 0.12^f^	12.18 ± 0.16^bc^	10.60 ± 0.07^e^	0.001	10.46^B^	11.53^A^	11.12^A^	10.46^B^	11.94^A^	10.04^B^	0.001	0.016	0.001	0.819	0.011	0.606	0.362
Unknown	0.87 ± 0.04	2.10 ± 0.19	2.21 ± 0.48	0.41 ± 0.22	2.22 ± 0.11	1.42 ± 0.59	1.43 ± 0.74	0.91 ± 0.10	2.43 ± 0.54	—	—	—	—	—	—	—	—	—	—	—	—	—	—

*Note:* Values (means of triplicate ± SE) in the same row sharing the same superscript letter are not significantly different (*p*  > 0.05).

Abbreviations: AP, animal by‐product protein; PP, plant protein; RL, replacement level.

^1^∑SFA, Total saturated fatty acids.

^2^∑MUFA, Total monounsaturated fatty acids.

^3^∑n‐3 HUFA, Total n‐3 highly unsaturated fatty acids.

## 4. Discussion

The improved growth performance, as reflected by higher WG and SGR, of fish fed the TBM‐containing diets compared with those fed the CBM‐containing diets indicates that TBM is a more effective replacer of FM in olive flounder feed. Likewise, olive flounders fed a diet replacing 25% of FM with a blend of TBM and plasma powder achieved superior growth performance compared to those fed a diet replacing 25% of FM with a blend of CBM and plasma powder [24]. Furthermore, Kim et al. [17] disclosed that olive flounder fed a TBM‐containing diet achieved superior WG and SGR compared to those fed a CBM‐containing diet when 30% of FM was replaced with various animal by‐product sources, such as TBM, hydrolyzed chicken offal meal, CBM, meat and bone meal, meat meal, and blood meal in a 65% FM basal diet. In addition, diets with a higher (50%) level of FM replacement resulted in inferior growth performance compared with diets with a lower (25%) level of FM replacement. This finding indicates that increasing dietary FM substitution levels generally deteriorates fish growth performance, which is consistent with previous studies [12, 14, 20, 22, 23, 28, 50–52] showing that higher levels of FM substitution were reported to depress the growth of fish. Higher, but not significantly higher, WG and SGR of olive flounder fed the TBCG25 and TBCP25 diets compared to those of fish fed the Con diet also indicate that 25% of FM substitution with a blend of TBM and CGM or CPC when supplemented with JMM as a feed enhancer can outperform the growth of fish fed a 60% FM‐based diet. Similarly, Choi et al. [41, 42] reported that replacing 20% and 40% of FM with the combined animal by‐product and plant protein sources (tankage meal, SPC, and poultry by‐product meal) in a 65% FM‐basal diet did not impair the growth of olive flounder.

The nutritional composition of a diet, particularly the AA profiles, is a critical factor to consider when replacing FM with alternative protein sources [53]. In this experiment, all experimental diets met the dietary demands of arginine, lysine, and threonine (2.04%–2.10%, 1.55%–1.97%, and 1.03%, respectively) for olive flounder [46, 47, 49]. However, the methionine content in all experimental diets including the Con diet appeared to be slightly lower than the reported methionine demand for this fish species (1.44%–1.49%) when 0.06% cysteine is present in a diet [48]. Cysteine is known to partially spare the methionine demand in fish, with sparing effects of approximately 50% and 60% reported in red drum (*Sciaenops ocellatus*) and channel catfish (*Ictalurus punctatus*), respectively [54, 55]. Although the relatively high cysteine levels in the experimental diets might have partially compensated for the marginally deficient methionine levels, cysteine cannot fully replace methionine when dietary methionine is insufficient [56]. Therefore, the relatively low methionine levels in the CBCG50 and CBCP50 diets might have contributed, at least in part, to the reduced growth performance observed in fish receiving these diets. Based on these findings, further studies are needed to evaluate whether dietary methionine supplementation can alleviate the growth reduction observed in fish fed the experimental diets under the present experimental conditions.

Marine carnivorous fish have evolutionarily adapted to diets naturally abundant in long‐chain n‐3 HUFAs, and these FAs are indispensable structural and functional components of cellular membranes, contributing to neural development, visual function, immune regulation, and overall physiological homeostasis [57]. When aquafeeds contain insufficient levels of ∑n‐3 HUFAs, fish may exhibit impaired growth performance, altered tissue lipid composition, and compromised health status [57–60]. In this experiment, all experimental diets, with the exception of the CBCG50 and CBCP50 diets, fulfilled the previously reported dietary demand for ∑n‐3 HUFAs in olive flounder (0.8%–1.0% of diet) [60], corresponding to approximately 6.89%–8.62% of total FAs. The reduced ∑n‐3 HUFAs in the CBCG50 and CBCP50 diets may; therefore, partially demonstrate the compromised growth performance of fish fed these diets relative to those receiving the Con diet. Higher levels of ∑n‐3 HUFAs in the TBM‐containing diets were associated with the intrinsically greater abundance of these FAs in TBM compared with those in CBM, CGM, and CPC.

In this experiment, fish fed the TBM‐containing diets exhibited significantly higher FC than those fed the CBM‐containing diets, suggesting that TBM serves as a more effective alternative to FM than CBM in the olive flounder diet, particularly in terms of feed palatability and growth‐related response. Similar positive impacts of TBM as a partial replacer have been reported in olive flounder [20] and spotted rose snapper (*Lutjanus guttatus*) [22].

Slightly, but not significantly, elevated FC in fish fed the TBCG25 and TBCP25 diets compared to that in fish fed the Con diet might be partially associated with their relatively higher histidine content (1.42%–1.43% of diet), whereas the comparatively lower histidine content (1.24%–1.25%) in the CBCG50 and CBCP50 diets producing poor growth coincided with reduced FC, with no significant differences in FC among dietary treatments. This finding aligns with the findings of Ikeda et al. [35], who demonstrated that specific AA, especially histidine, acts as a potent feeding stimulant in olive flounder. Feeding behavior is regulated through two primary chemosensory mechanisms: olfactory perception, which facilitates feed detection and localization, and gustatory perception, which influences ingestion [61, 62]. AAs, such as methionine, lysine, proline, glycine, and alanine, are also recognized as key chemical cues capable of stimulating feeding responses in yellow river carp (*Cyprinus carpio*) [63]. Moreover, increasing dietary FM replacement levels led to lower FC compared with diets containing lower FM replacement levels in the current experiment. This was consistent with other studies, in which higher dietary FM substitution levels with alternative protein sources reduced feed palatability, limited voluntary feed intake, and ultimately impaired the WG of fish [11–13, 18].

Although the main effects of the animal by‐product source and FM replacement level significantly influenced FC, comparisons of the individual diets with the Con diet revealed no significant differences in FC. In fish fed the TBM‐containing diets, this response may be partly associated with the relatively high contribution of marine‐origin protein and lipid sources, including TBM, JMM, and fish oil, as marine‐origin protein sources are generally associated with improved feed palatability in aquafeeds [64]. In addition, it is noteworthy that fish fed the CBM‐containing diets maintained FC comparable to that of the Con diet. However, the poorer growth responses observed in fish fed the CBCG50 and CBCP50 diets compared with those fed the Con diet indicate that maintaining FC alone was not sufficient to sustain an appropriate growth performance. Therefore, the reduced growth performance in these diets may not be explained solely by differences in FC but may also be associated with other nutritional factors, such as ingredient combination, nutrient availability, AA balance, nutrient digestibility, or FM replacement level [64, 65].

Feed utilization (FE, PER, and PR) and biological indices (CF and HSI) of olive flounder, with the exception of a significant impact of animal by‐product source on VSI, were not statistically influenced by either dietary animal by‐product source, plant protein source, or FM substitution levels in the present experiment. Moreover, FE, PER, PR, CF, and HSI of fish fed the FM‐replaced diets were not significantly different from those of fish fed the Con diet, indicating that FM substitution did not impair these parameters. Similarly, Jeong and Cho [66] reported no significant differences in feed utilization and biological indices when olive flounder were provided with diets substituting 25% and 50% of FM with TBM, CBM, or meat meal, respectively. Choi et al. [42] also found that dietary 20%–40% of FM replacement with blended protein sources (poultry by‐product meal, SPC, and tankage meal) did not affect PER, CF, VSI, and HSI of olive flounder but significant decline in FE at the 40% FM replacement level.

Blood plasma parameters serve as the dependent indicators for monitoring physiological well‐being and nutritional health of fish [67], and analyzing these parameters within a consistent experimental framework allows for a robust evaluation of dietary impacts [68]. In this study, no significant differences in plasma parameters were observed between fish fed the FM‐replaced diets and those fed the Con diet. In addition, neither dietary animal by‐product, plant protein source, nor FM substitution levels negatively altered plasma parameters, suggesting that olive flounder maintained optimal physiological conditions regardless of dietary treatments. Similarly, Gunathilaka et al. [25] showed no remarkable changes in plasma parameters in red sea bream fed diets replacing 50% of FM with SPC, CGM, meat meal, and CBM or their blends. In contrast, Lu et al. [69] observed significant changes in plasma GOT in rainbow trout fed diets replacing 75% and 100% of FM with blended animal and plant protein sources. Similarly, significant fluctuations in plasma parameters were reported in olive flounder [11] and spotted rose snapper [28] when FM was replaced by CGM or TBM in their diets.

Serum lysozyme serves as a fundamental component of the innate immune system, defending against pathogens and toxins, while SOD acts as a critical antioxidant enzyme that mitigates oxidative stress in fish [70]. Therefore, comparisons of serum lysozyme activity and SOD levels can provide valuable insights into their overall health and immune status [71]. In this study, no significant differences in serum lysozyme activity or SOD levels were observed between fish fed the FM‐replaced diets and those fed the Con diet. In addition, the lack of any pronounced differences in these variables suggests that neither dietary animal by‐product source, plant protein source, nor FM replacement level influenced the innate immunity of olive flounder. Similarly, Choi et al. [42] showed no statistical differences in serum lysozyme activity and SOD levels in olive flounder fed diets replacing 40% of FM with blended animal by‐product and plant protein sources. In contrast, Gunathilaka et al. [25] observed a stimulation of lysozyme activity in red sea bream fed a diet replacing 30% of FM with a blend of animal by‐product and plant protein sources. Such discrepancies may be attributed to the fact that lysozyme activity is highly sensitive to both species‐specific responses and dietary formulations as certain ingredients possess the capacity to modulate these immune‐related responses [72].

No significant differences in the chemical composition and AA profiles of whole‐body fish were observed among fish fed all experimental diets. In addition, these parameters were not affected by dietary animal by‐products, plant protein sources, or FM replacement levels. Likewise, the proximate composition and AA profiles in olive flounder were not affected by dietary FM replacements with various animal by‐product (CBM, TBM, and meat meal) [67] and plant protein sources (CGM, SPC, and CPC) [30]. In addition, dietary FM replacement with various alternative sources and levels did not bring about any statistical differences in the chemical composition and AA profiles in red sea bream [25] and rockfish [31]. There are also some contracting results, in which dietary FM replacement with animal by‐product or plant protein sources affected the proximate composition in olive flounder [19] and red sea bream [73], as well as the AA profiles in olive flounder [74] and black sea bream (*Acanthoparus schlegelii*) [75].

Fish fed the CBM‐containing diets led to higher whole‐body ∑MUFAs but lower ∑n‐3 HUFAs compared to those fed the TBM‐containing diets in this study. The highest level of ∑n‐3 HUFAs was observed in fish fed the Con diet. These changes in FA profiles in the whole‐body fish were well‐driven from dietary FA profiles, agreeing with other studies [17, 26, 60, 76], in which the FA profiles of fish were directly associated with dietary FA profiles. In addition, increasing dietary FM substitution levels led to increased whole‐body ∑MUFAs but decreased ∑n‐3 HUFAs. This is coincident with other studies [50, 52], where dietary increased FM replacements with alternatives led to elevated ∑MUFAs but decreased ∑n‐3 HUFAs in the whole‐body fish.

## 5. Conclusion

The TBM‐containing diets exhibited superior outcomes in growth performance and FC compared to those of the CBM‐containing diets. In addition, dietary lower (25%) FM substitution levels outperformed dietary higher (50%) FM substitution levels. TBM seemed to be a more compatible animal by‐product source than CBM when combined with plant protein sources (CGM and CPC) as a replacer for FM in the olive flounder diet. Notably, fish fed the TBCG25 and TBCP25 diets showed higher, but not significantly higher, growth performance and FC than those fed a 60%‐FM basal diet. Therefore, the TBCG25 and TBCP25 diets seemed to be the most recommendable strategies for olive flounder farmers. To confirm; however, feasibility of these diets should be validated in commercial‐scale farm before widespread industry adoption.

## Author Contributions

**Seong Woo Shin**: investigation, data curation, writing – original draft. **Md. Rabiul Islam**: data curation, writing – original draft. **Sung Hwoan Cho**: methodology, conceptualization, funding acquisition, writing – review and editing. **Taeho Kim**: funding acquisition.

## Funding

This research was supported by NRF grant funded by the Korean government (Grant RS‐2026‐25477661). This research was supported by the Korea Institute of Marine Science & Technology Promotion (KIMST), funded by the Ministry of Oceans and Fisheries (Grant RS‐2026‐25544101).

## Ethics Statement

The feeding trial and subsequent handling and sampling of experimental fish were carried out as per the ethical guideline of the Korea Maritime and Ocean University (KMOU IACUC 2026‐03).

## Conflicts of Interest

The authors declare no conflicts of interest.

## Data Availability

Data are available upon request from the authors.
